# A pilot comparison of phospolipidated lutein to conventional lutein for effects on plasma lutein concentrations in adult people

**DOI:** 10.1186/s12937-015-0089-x

**Published:** 2015-10-07

**Authors:** Robert A. DiSilvestro, Sara Thomas, Earl Harrison, Alice Epitropoulos

**Affiliations:** 1 Columbus Nutraceutical Formulations LLC, 8050 Simfield Rd, Dublin, OH 43016 USA; 2 Human Nutrition, Ohio State University, Columbus, OH USA; 3 OhioHealth, Columbus, OH USA

**Keywords:** Lutein, Absorption, Human, Solid-lipid particle, Supplementation, Carotenoid

## Abstract

**Background:**

The percent absorption of lutein from supplements falls well below that from lutein enriched egg yolk, a rich source of phospholipids. Therefore, a supplement of lutein plus phospholipids was tested for effects on serum accumulation.

**Methods:**

A 10 day supplementation with a solid-lipid particle (SLP*™*) lutein complex or conventional lutein ester was done in apparently healthy people (both supplement types taken with fat containing meals). Plasma lutein was measured pre- and post-supplementation as well as 7 days after supplementation stopped. Changes within each supplement group were analyzed by paired *t*-test; group comparisons were done by unpaired *t*-test.

**Results:**

The solid-lipid particle complex lutein gave much higher plasma lutein values than conventional lutein ester. The lutein complex showed superior effects based on absolute mean value after 10 days of supplementation, change in value from baseline to 10 days, and value at 7 days after supplement discontinuation.

**Conclusions:**

A solid lipid lutein complex strongly increased plasma lutein levels compared to a conventional form.

## Introduction

Lutein, a member of the carotenoid family, is a non-essential nutrient that has shown ability to accumulate in the eye, perform antioxidant actions relevant to protection of the eye, affect macular pigment density, and improve multifocal electroretinogram response in people with macular degeneration (reviewed in [1]). Also, diets high in carotenoids that include lutein show an inverse correlation with aging related eye problems (reviewed in [1]).

Lutein can be obtained from foods as well as from nutritional supplements. Lutein ester from enriched egg yolk has displayed superior absorption than lutein from certain vegetable or supplement sources [2]. In a later study [3], a 90 day intake of 1 mg/day of lutein in enriched eggs is claimed to produce the same serum lutein as 5 mg of lutein as a supplement.

Although the amount of lutein per serving of normal eggs falls far below that of a few vegetable sources, egg consumption have been found to raise serum lutein readings [4, 5]. Presumably, this effect of eggs occurs due to the strong absorption of lutein ester from this source. These observations raise the question: Can an especially effective supplement be made by complexing lutein with phospholipids and fatty acids that overlaps those of eggs? Therefore, a pilot study was conducted to evaluate plasma accumulation of such a complex compared to a conventional lutein.

## Methods and materials

The study protocol was approved by the OhioHealth Institutional Review Board. All subjects signed an informed consent form. Subjects were 12 males and females (six of each gender) aged 52 to 69, mean ± SD of 57 ± 3 for the standard lutein, and 59 ± 6 for the novel lutein complex. Based on answers to an eligibility questionnaire, the accepted subjects were nonsmokers who were free from problems that cause widespread oxidant stress or cause problems with absorption of lipid nutrients. Also, based on answers to the questionnaire, subjects did not consume eggs, spinach, or kale more than four times a month, nor take lutein supplements.

Subjects were randomly assigned to either lutein ester or a solid-lipid particle (SLP*™*) complex lutein. The latter was supplied by Verdure Sciences, Noblesville, IN, USA. The subjects took a single capsule of 10 mg lutein for 10 days (same mg of lutein/day/treatment, though different weights of total powder). Subjects were blinded to group assignment. The capsules were taken with a self-selected meal containing at least 200 Calories of fat. The subjects provided a blood sample in a heparin containing tube before and after the 10-day supplementation period as well as 7 days after discontinuing the supplement. Plasma was separated by centrifugation for 30 min at 3000 rpm. Plasma lutein was determined by HPLC [6].

Changes within each supplement group were analyzed by paired *t*-test using http://www.fon.hum.uva.nl/Service/Statistics/Student_t_Test.html Group comparisons were done by unpaired *t*-test using http://www.fon.hum.uva.nl/Service/Statistics/2Sample_Student_t_Test.html

## Results

**Fig. 1:**
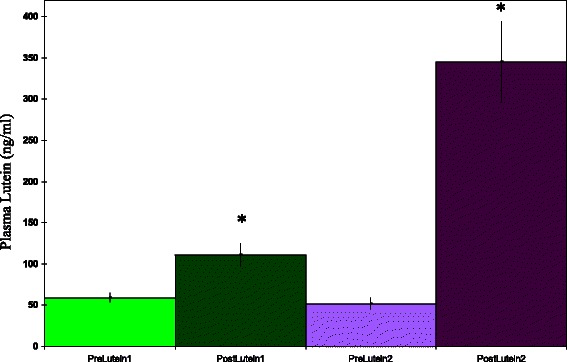
Plasma lutein concentrations before and after 10 days supplementation with 10 mg/day of lutein. Lutein 1 = lutein ester. Lutein 2 = solid-lipid particle complex lutein. *Significantly different from pre value, *p* < 0.001, paired *t*-test

**Fig. 2:**
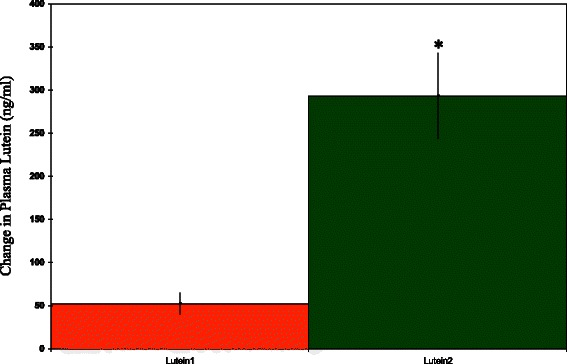
The change in plasma lutein concentrations after 10 days of supplementation with 10 mg/day of lutein. Lutein 1 = lutein ester. Lutein 2 = solid-lipid particle complex lutein. *Significantly different from pre value, *p* < 0.002, unpaired *t*-test

**Fig. 3:**
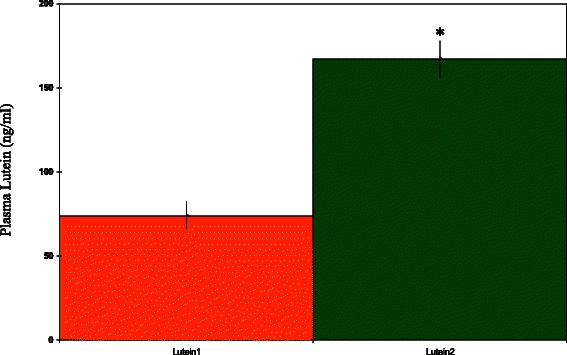
Plasma lutein concentrations 7 days post-supplementation (10 mg/day of lutein for 10 days). Lutein 1 = lutein ester. Lutein 2 = solid-lipid particle complex lutein. *Significantly different from Lutein1 value, *p* < 0.001, unpaired *t*-test

After 10 days of supplementation, both supplements produced highly significant increases in plasma lutein values (Fig. 1, *p* < 0.001 for each treatment, paired *t*-test). The solid-lipid particle complex lutein gave a much higher mean plasma lutein value than conventional lutein (*p* < 0.001, unpaired *t*-test). The mean percent change versus pre-supplement values was 563 % for the solid-lipid particle complex lutein and 88 % for the conventional lutein ester. If the data was expressed as the change in lutein concentrations, a much higher mean change was seen with the solid-lipid particle complex (Fig. 2, *p* < 0.001, unpaired *t*-test). For both the conventional and new lutein supplement, mean plasma lutein levels remained above baseline 7 days after supplementation (Fig. 1, pre-values vs Fig. 3, *p* < 0.001, paired *t*-test). However, the solid-lipid particle complex lutein gave a much higher mean plasma lutein value (Fig. 3, *p* < 0.001, paired *t*-test). Thus, by three types of evaluations, plasma lutein concentrations responded to a much greater degree to the solid-lipid particle complex lutein than to a conventional version.

## Discussion

In this pilot study, solid-lipid particle complex lutein clearly produced much higher mean plasma lutein values than a conventional lutein. Admittedly, a small subject number was examined. However, even for this small number, impressively low p values were obtained for the various comparisons. Moreover, the quantitative differences reached high proportions and the results were very consistent among all participants.

In this study, the conventional lutein consisted of an ester. In one comparison with free lutein, an ester fares better [7]. In contrast, in two other studies [8, 9], free lutein produced a better serum response than an ester, at least for some types of comparison. However, even if the favorable results for free lutein are considered, the percent differences for free versus ester don’t come close to the present study’s results for solid-lipid particle complex versus ester. Thus, the solid-lipid particle complex lutein would likely show much stronger effects than free lutein.

The higher plasma lutein concentrations produced by the solid-lipid particle complex lutein is assumed to reflect better absorption from the GI tract. However, in theory, the high plasma values could instead reflect poor uptake into body tissues. Two lines of reasoning make this unlikely. First, no studies report this type behavior, but studies have shown increases in serum or plasma lutein to be accompanied by tissue increases (ie [10–12]). Second, if this study’s results are due to poor tissue uptake of the solid-lipid particle complex lutein, this complex would have to enter intestinal cells well, but not enter other types of cells well. Nothing in current knowledge of carotenoid metabolism points to the possibility of such behavior. It can be further noted that in many studies on relative lutein absorption, plasma or serum lutein levels are used as the endpoint (ie [2–5, 7–10]).

A question that could be raised about the current results is: Do the high plasma lutein concentrations obtained in the present study translate to better eye health? This cannot be answered yet, but some data suggests such high concentrations can promote eye health. In a study on lutein ester supplementation [10], going from 5 to 10 to 20 mg/day increases both serum lutein readings and macular pigment optical density. Thus, no evidence was found that macular pigment optical density peaks at the rises in serum lutein seen in that study. However, for serum lutein readings in that study, the fold differences between 10 and 20 mg lutein ester fell way below the fold differences seen here for 10 mg lutein ester versus solid-lipid particle complex lutein. Thus, it can be projected that 10 mg of phospolipidated lutien would likely more strongly impact macular pigment optical density than the 20 mg lutein ester used in the previous work. Therefore, it could be projected that the 10 mg of solid-lipid particle complex lutein could likely translate into higher macular pigment optical density readings than given by the 20 mg of lutein ester studied earlier.

A comparison between the current study and the just discussed previous study [10] cannot be made based on absolute values for serum or plasma lutein. Such values rose much higher in the previous study, but the previous study used a much longer intervention time.

One question that can arise about the solid-lipid particle complex lutein is whether the dose of the present study can produce toxicity. Rodent studies provide evidence against such a possibility. Even when serum lutein is greatly increased by tremendously high lutein doses, toxicity is not found [13, 14]. Also, in rabbits, high elevation of eye lutein levels by direct lutein application does not cause toxicity [15].

In summary, a 10 day supplementation of solid-lipid particle complex lutein produced far greater plasma accumulation than lutein ester. The solid-lipid particle complex lutein merits consideration for possible use in eye health supplements.
